# Spatial and temporal pattern evolution and driving characteristics of rural settlements in Xilingol League from 1915 to 1980

**DOI:** 10.1371/journal.pone.0336034

**Published:** 2026-04-10

**Authors:** Sen Mu, Jianghong Zhen, Chun Xi

**Affiliations:** 1 College of Geographical Sciences, Inner Mongolia Normal University, Hohhot, Inner Mongolia Autonomous Region, China; 2 Key Laboratory of Mongolian Plateau Climate Change and Regional Response, Inner Mongolia Normal University, Hohhot, Inner Mongolia Autonomous Region, China; Hunan University, CHINA

## Abstract

Population migration and agricultural expansion have profoundly reshaped the rural characteristics of Xilingol League, a typical pastoral region in northern China. From the perspective of historical geography, this study investigates the settlement evolution in Xilingol League during 1915–1980. Drawing on declassified statistical data and historical documents, and employing kernel density estimation, hotspot analysis, and the geographical detector, we reconstruct and interpret the settlement patterns during the period without remote sensing imagery. The results reveal that: (1) Between 1915 and 1980, settlement expansion in Xilingol League exhibited a fan-shaped trajectory centered on Taibus Banner and Duolun County, with clustering intensity diminishing with increasing distance. (2) The 42°N parallel demarcates pastoral areas from agro-pastoral transitional zones, with settlements concentrated mainly in the transitional zone and showing a fragmented northeastward expansion into pastoral areas over time. (3) Socio-economic conditions were the dominant drivers of settlement evolution during this period, with cultivated land area (*q* = 0.6266) and livestock numbers (*q* = 0.6215) exerting the strongest explanatory power. This study provides new insights into the processes and driving mechanisms of modern settlement evolution in Xilingol League.

## 1. Introduction

Settlements are places where people reside, rest, and engage in diverse social activities, serving simultaneously as centers of production and daily life [1]. The theoretical framework for settlement studies was first established by Christaller through his central place theory, which sought to explain the spatial distribution patterns and service functions of settlements in southern Germany [2]. This classical theory remains applicable to the settlement patterns of semi-arid regions, where the origins of nomadic economies are also found. Constrained by the natural environment, these areas gave rise to mixed agro-pastoral systems that integrate livestock husbandry with crop cultivation in rural settings [3]. In recent years, international scholarship on rural and pastoral systems has primarily focused on settlement ecology, spatial analysis, and landscape studies [4]. Dibissa [5] examined the mixed farming systems of the Ethiopian highlands, noting that cropland size and food production are constrained mainly by land ownership, while livestock production depends heavily on communal grazing lands. Sturaro [6] revealed that the interwoven nature of rural settlements and pastures in the Alpine region contributes to high variability in mountain pastoral systems. Calafat-Marzal [7] emphasized the importance of spatial planning in pastures to mitigate regulatory and environmental risks associated with livestock expansion and population concentration. Milojevic [8] applied quantitative methods to classify Serbian pastures into three types, thereby highlighting the diversity of local pastoral production systems. Overall, agricultural and pastoral systems exhibit high levels of diversity, with pastoralism functioning across multiple dimensions and reflecting the historical, biological, social, and ethnographic characteristics of specific regions [9]. Today, urbanization has driven agricultural and pastoral systems in nearly all regions toward more intensive, market-oriented models, and the interaction between urbanization and the pastoral economy has become a mainstream topic of research [10].

In the process of rapid industrialization and urbanization, the roles and functions of agricultural and pastoral areas—as well as their position within society—have been undergoing constant transformation [11,12]. Since the beginning of the 21st century, settlement studies in China have progressed through three distinct stages: initiation, development, and maturity. During this process, research topics and perspectives have gradually diversified, while related rural policy studies have evolved from initial exploration to further consolidation and, ultimately, to a stage of deepening [13]. Building on traditional agricultural geography, Chinese rural geographers have increasingly taken economic and social transformation as the central thread, thereby enriching and extending research from agricultural production systems to broader rural regional systems [14]. This growing body of research provides an important academic foundation for understanding the dynamics of rural change, while also highlighting the need for more nuanced approaches that can capture the complexity of local contexts. Against the backdrop of contemporary rural revitalization, a better understanding of future rural reform trends requires research approaches that are more targeted, differentiated, and context-sensitive [15].

With the advancement of GIS technologies, some historical geographers have begun to employ modern spatial tools to investigate the evolution and transformation of settlements [16,17]. Current research by scholars mainly focuses on the distribution [18], morphology [19], evolution [20], and driving factors of settlements [21]. Through toponymic data, have been able to reveal, to some extent, the evolutionary process of settlements across regions on a long time scale [22],They have continually broadened their research perspectives, analyzing settlement evolution processes in various regions from different angles, such as mountain settlements [23], and southern China water towns [24]. In recent years, the improvement of geographic information data has provided numerous conveniences for monitoring [25], evolution [26] and detection [27] of geographical environments on long time scales. The enhancement of technological methods has laid an important foundation for subsequent rural revitalization research and provided pathways for the implementation of rural protection plans [28]. However, most of these studies have focused on agricultural regions characterized by continuous historical records and dense populations. In contrast, due to differences and barriers in language, written documentation, and living practices, relatively limited attention has been given to the evolution of settlements in pastoral and agro-pastoral transitional areas.

Pastoral nomadism is a production and management system centered on livestock husbandry, characterized by continuous migration across vast territories to sustain herd supply. Consequently, it exhibits substantial differences in spatial organization compared to the settlement patterns of agricultural and sedentary societies [29]. Zhou and Wang, drawing on settlement data from 1990 to 2020, analyzed the evolution of settlements in Inner Mongolia and found that their spatial distribution was significantly shaped by factors such as proximity to arable land, distance from towns, and population density [30]. Moreover, Inner Mongolia spans a vast east–west extent, and striking socio-economic disparities between its eastern and western regions have attracted considerable scholarly attention [31]. Xilingol League is located in central Inner Mongolia and has vast natural grasslands, with its pastoral culture significantly influencing local production and life. To better understand the historical evolution of settlements in Xilingol League between 1915 and 1980, this study constructs a triadic analytical framework of institution–economy–space, embedding the discussion within the broader historical context of land reform, ethnic policies, and state-led migration. At the institutional level, state-led land reforms, sedentarization programs for herders, and property rights arrangements provided the institutional foundation for the formation and spatial distribution of settlements [32]. Li and Ali discussed the household responsibility system, arguing that it stimulated the development of animal husbandry [33], and further recommended promoting small-scale collective property systems in Xilingol’s pastoral areas [34]. At the economic level, with the transformation of agricultural and pastoral production systems and the continuous increase in market demand, the growing global appetite for livestock products has driven producers to expand animal and feed production, resulting in substantial land-use changes [35]. Improvements in transportation and infrastructure further contributed to the marked growth and restructuring of settlement numbers and scales [36]. At the spatial level, natural geographical conditions—such as elevation, slope, water resources, and grassland distribution—interacted with locational and transportation factors [37], collectively shaping the spatial configuration and expansion trajectories of settlements [38]. Meanwhile, climate change has also emerged as an important driver influencing herders’ transition toward sedentarization [39].

While many scholars have examined land use, the development of animal husbandry, and their impacts on urbanization in Xilingol League, research on settlement evolution prior to the reform and opening-up period remains limited. By systematically applying the institution–economy–space triadic framework and analyzing their interactions, and by integrating statistical data with historical documents, this study aims to explore the settlement evolution of Xilingol League between 1915 and 1980, building on previous research by local scholars [16,40–43]. This approach not only uncovers the underlying mechanisms that drove the transition from a traditional nomadic society to sedentarization and modernization in the region, but also provides an in-depth analysis of the evolutionary processes and driving factors. More broadly, it offers a methodological perspective for studying settlement evolution in pastoral and agro-pastoral areas where the sedentarization process is not well defined and historical records are often fragmented.

## 2. Study area and methods

### 2.1. Study area

**Fig 1:**
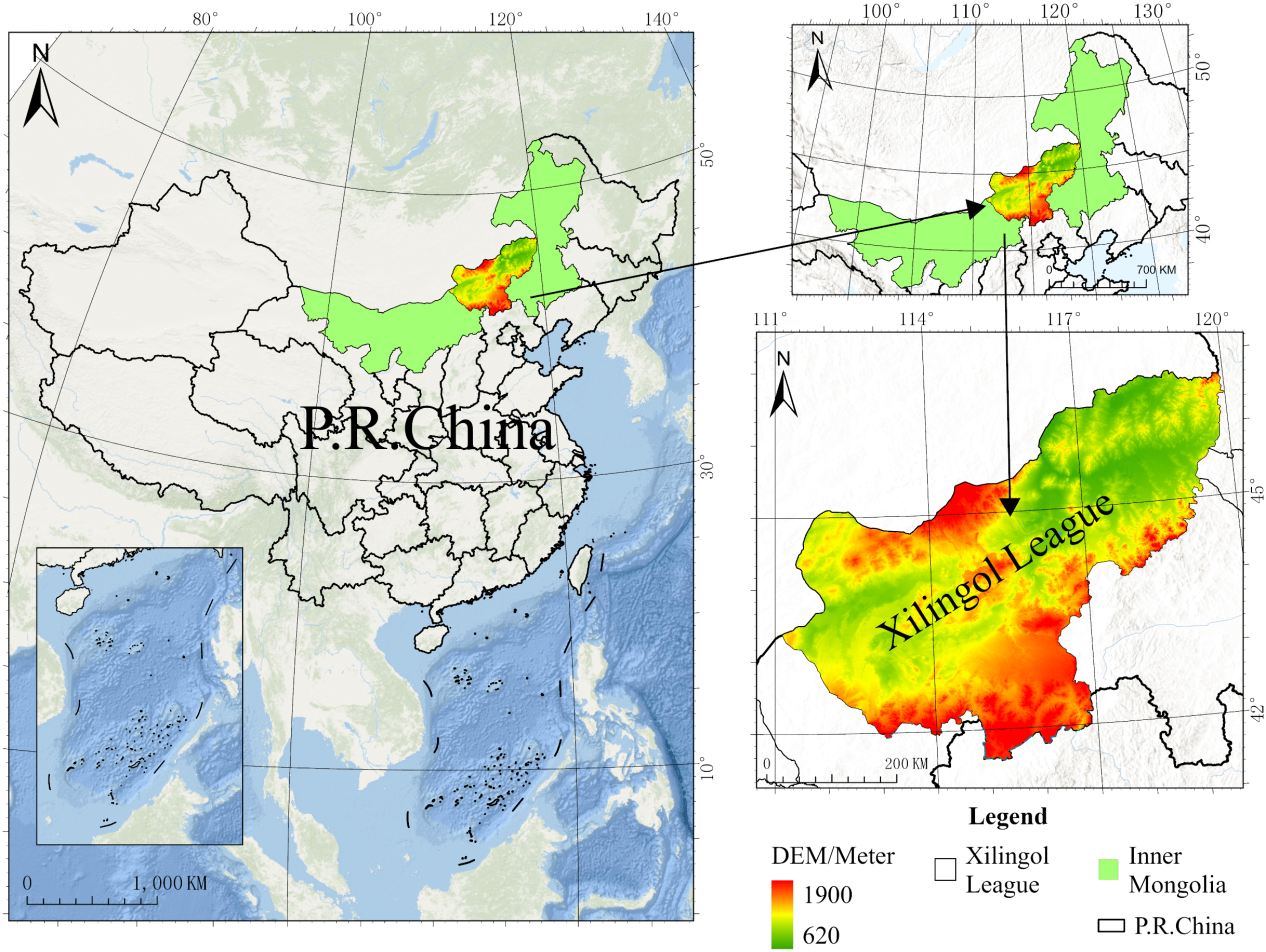
Location of the study area. The map is produced based on the standard map from the Ministry of Natural Resources of China (No. GS(2019)1652), with no modification to the base map.

Xilingol League is located in central Inner Mongolia Autonomous Region, spanning latitudes $42^{\circ }32^{\prime }$ to $46^{\circ }41^{\prime }$ N and longitudes $111^{\circ }59^{\prime }$ to $120^{\circ }00^{\prime }$ E (Fig 1). It has an administrative area of 202,600 km², comprising 2 county-level cities, 9 banners, 1 county, and 1 administrative district. The league has an average annual temperature of 3.5°C and an average annual precipitation of 276.5 mm. In 2023, its regional GDP reached 118.478 billion yuan, and by the end of 2023, the permanent resident population was 1.1165 million. This study covers a total of 1,854 settlements.

Xilingol League encompasses approximately 179,600 km² of meadow steppe, 20 major river systems, and over 470 lakes, with a total water surface area of about 500 km². These favorable natural conditions have long sustained it as a traditional Mongolian habitation area and a representative pastoral region, where the nomadic practice of “following water and grass” persisted for centuries. Since the founding of the People’s Republic of China, however, improvements in productivity, industrialization, the siphoning effect of urbanization, and the implementation of grassland conservation policies have collectively driven a transition from nomadic to sedentary livelihoods among pastoralists. At present, Xilingol League consists of 10 predominantly pastoral banners (Xilinhot, Erenhot, Sonid Left Banner, Sonid Right Banner, Abaga Banner, East Ujimqin Banner, West Ujimqin Banner, Plain and Bordered White Banner, Plain Blue Banner, and Bordered Yellow Banner) and 2 agro-pastoral transitional banners/counties (Taibus Banner and Duolun County). The coexistence of mixed agro-pastoral production systems and diverse ethnic groups has shaped the distinctive spatial distribution and historical evolution of settlements across the region.

### 2.2. Data sources

**Fig 2:**
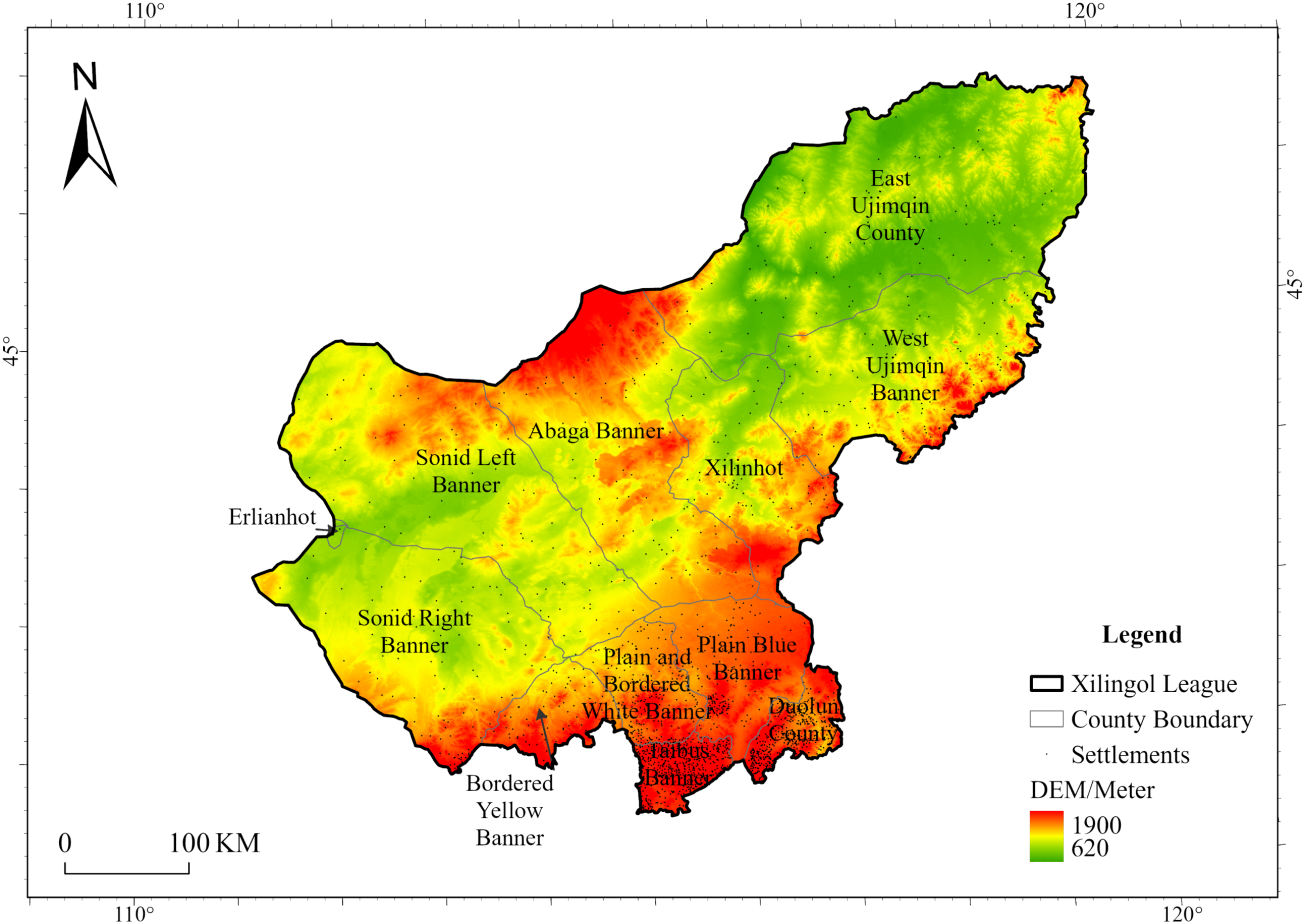
Settlement distribution in Xilingol League in 1980. The map is produced based on the standard map from the Department of Natural Resources of Inner Mongolia (No. MS(2023)039), with no modification to the base map.

The settlement data were obtained from the 1:50,000-scale vector dataset of settlement points in Inner Mongolia, provided by the Inner Mongolia Department of Natural Resources. Through field surveys, local chronicles, and other historical records, the establishment dates of various settlements were documented. Using 1980 as the temporal cutoff, a total of 1,834 settlement points were selected (Fig 2). The DEM data were sourced from the Geospatial Data Cloud of the Chinese Academy of Sciences (https://www.gscloud.cn) at a resolution of 30 meters. ArcGIS Pro software was used to extract elevation and slope data for the settlement points. Mean annual temperature and annual precipitation data were obtained from the Earth Resources Data Cloud (http://www.gis5g.com) at a resolution of 1 kilometer. The vector boundary data were obtained from the Resource and Environment Science and Data Center (RESDC) of the Chinese Academy of Sciences, and the map review number was sourced from the official website of the Inner Mongolia Department of Natural Resources. The boundary range has not been altered (https://www.resdc.cn/DataList.aspx). Socioeconomic data for each banner and county were obtained from legally declassified data in the Xilingol League Statistical Yearbook, provided by the Xilingol League Bureau of Statistics.

### 2.3. Method

First, data preprocessing was conducted to determine the establishment time of each settlement. Settlement construction years were identified by consulting the National Database for Geographical Names of China (https://dmfw.mca.gov.cn/search.html), the Gazetteer of Xilingol League, individual banner gazetteers, and field interview records. In addition, historical sources such as the Draft Gazetteer of Suiyuan, the Gazetteer of Xilingol League, the Gazetteer of Ulanqab, and the Gazetteer of Zhangjiakou were used to reconstruct the administrative evolution of the region. The settlement establishment years were then compiled into a database.

The study period was divided according to key historical events that significantly influenced settlement change, with each interval spanning approximately 10–15 years. Specifically, the temporal segmentation was defined as follows: the land reclamation initiated by Mongolian nobles in 1915;the Japanese invasion of Chahar and the establishment of the puppet Mongolian government in 1933; the founding of the People’s Republic of China and land reform in 1949; the People’s Commune Movement in 1958; the adjustment period of the Cultural Revolution and the large-scale “sent-down youth” movement in 1970; and the onset of reform and opening-up in 1980, when satellite imagery records also became available. Based on these temporal divisions, settlements were screened by period to obtain their number and spatial distribution, and settlements outside the defined timeframe were removed. In total, 1,834 settlement points were retained for analysis.

#### 2.3.1. Kernel density estimation method.

The kernel density estimation (KDE) method is employed to calculate the density of point or line features within a defined neighborhood. This approach provides an intuitive visualization of settlement distribution density and effectively captures the spatial characteristics of settlement patterns in the study area. It also allows for a precise assessment of the concentration and intensity of settlement distribution [13]. Moreover, KDE can infer the overall distributional characteristics from sample data and has been widely applied in analyzing the spatial distribution and variation patterns of different land-use types [44]. The general formula for kernel density estimation is expressed as follows:


$$f(x)=\sum_{i=1}^{n}K(\frac{x-x_{i}}{h}),$$ (1)

where *f*(*x*) represents the kernel density value, K(·) denotes the kernel function, *h* is the bandwidth which defines the spatial extent around settlement points, *n* is the number of settlements within the defined range.

#### 2.3.2. Mean center.

The primary objective is to determine the central location of settlement distribution across different periods within the study area. This method calculates the mean center by averaging the and coordinates of all features in the region [45]. The formula is expressed as follows:


$$\bar{X}=\frac{\sum_{i=1}^{n}x_{i}}{n},$$ (2)

$$\bar{Y}=\frac{\sum_{i=1}^{n}y_{i}}{n}.$$ (3)

$\bar{X}$ and $\bar{Y}$ represent the mean center coordinates, *x*_*i*_ and *y*_*i*_ are the coordinates of individual settlement points, *n* is the total number of settlements within the study area.

#### 2.3.3. Standard deviation ellipse.

The standard deviational ellipse (SDE) is applied to characterize the spatial attributes of geographic features, including central tendency, dispersion, and directional trends [24]. The resulting ellipse illustrates both the spatial extent and the orientation of the data distribution. The ellipse center corresponds to the centroid of the settlement pattern, while the major axis reflects the dominant orientation of settlement distribution across the study area. The minor axis indicates the degree of dispersion perpendicular to the dominant direction. A larger disparity between the major and minor axes denotes a stronger directional trend, whereas a smaller disparity suggests a more scattered distribution with weaker directionality. When the two axes are equal, the ellipse becomes circular, indicating the absence of a distinct directional trend. The calculation formulas for the standard deviational ellipse are given as follows:


$$SDE_{X}=\sqrt{\frac{\sum_{i=1}^{n}{\left(x_{i}-\bar{X}\right)}^{2}}{n}},$$ (4)

$$SDE_{Y}=\sqrt{\frac{\sum_{i=1}^{n}{\left(y_{i}-\bar{Y}\right)}^{2}}{n}}.$$ (5)

*SDE*_*X*_ and *SDE*_*Y*_ are the standard deviations along the *X* and *Y* axes, respectively. *x*_*i*_ and *y*_*i*_ are the coordinates of each settlement, $\left\{\bar{X},\bar{Y}\right\}$ are the mean center coordinates, *n* is the total number of settlements.

#### 2.3.4. Hot spot analysis.

Hotspot analysis is a spatial statistical method used to evaluate and identify clustering relationships within the framework of local spatial autocorrelation. It measures the degree of association between each observation unit and its neighboring units [46]. This method plays a critical role in analyzing spatial distribution patterns and exploring spatial autocorrelation, offering advantages such as detecting spatial autocorrelation, identifying hotspots and cold spots, and assessing statistical significance [47]. The statistical metric employed in this analysis is the Getis-Ord index, which is calculated as follows:


$$G_{i}^{*}=\frac{\sum_{j=1}^{n}w_{ij}x_{j}-\bar{x}\sum_{j=1}^{n}w_{ij}}{s^{2}},$$ (6)

where *x*_*j*_ is the attribute value of feature *j*, *w*_*ij*_ is the spatial weight between features *i* and *j*, $\bar{X}$ is the mean of all feature values, *s* is the standard deviation of all feature values, *n* is the total number of features.

#### 2.3.5. Average Nearest Neighbor analysis.

The Average Nearest Neighbor (*ANN*) Analysis is used to calculate the mean distance between each feature and its nearest neighboring feature centroid. It is a commonly used index to assess the spatial proximity of point features. The mathematical expression for is as follows:


$$ANN=\frac{r_{1}}{r_{E}},$$ (7)

$$r_{E}=\frac{1}{2\sqrt{\frac{A}{n}}}=\frac{1}{2\sqrt{D}}.$$ (8)

Where, *ANN* denotes the Average Nearest Neighbor Index. An *ANN* value greater than 1 indicates a dispersed distribution of settlements, where settlements are spaced farther apart. *ANN* value less than 1 suggests a clustered distribution, with settlements concentrated in space. *ANN* value equal to 1 implies a random distribution. Moreover, the smaller the *ANN* value, the higher the degree of clustering in the settlement pattern. In this analysis, *n* represents the total number of settlements, *A* refers to the total area of Xilingol League, and *D* denotes the sample density. By applying this method, the overall spatial distribution characteristics of settlements in the study area can be quantitatively assessed, providing insights into settlement layout trends.

#### 2.3.6. Imbalance Index.

The Imbalance Index is used to measure the degree of harmony in resource allocation across different regions. In modern geographical research, it quantifies the uniformity of settlement distribution within various areas. The formula for the Imbalance Index is as follows:


$$S=\frac{\sum_{i=1}^{n}Y_{i}-50(n+1)}{100n-50(n+1)},$$ (9)

where *n* represents the total number of settlements in Xilingol League, *Y*_*i*_ denotes the cumulative percentage at the *i*-th position. The cumulative percentage for each region is ranked in descending order based on its proportion of the total number of settlements in the league. *S* ranges between 0 and 1, serving as an indicator of settlement distribution balance: *S*=0 indicates an even distribution of settlements across all regions; *S*=1 indicates that all settlements are concentrated in a single region, representing extreme imbalance.

#### 2.3.7. Geodetector.

The Geographical Detector (GeoDetector) model, originally proposed by Wang Jingfeng [48], is a spatial statistical tool designed to reveal the spatial heterogeneity of geographic phenomena and identify their key influencing factors. This method has been widely applied in studies of settlement spatiotemporal evolution to simulate the driving forces behind settlement pattern changes. It is suitable for diverse geographic environments and effectively avoids multicollinearity issues in the calculation process [49–51].

In this study, based on data availability and scientific rigor, indicators were selected from multiple dimensions—including natural, economic, and ecological factors—drawing on existing literature while also accounting for the regional context. The GeoDetector model was then employed to identify the primary factors influencing settlement evolution in Xilingol League.

Specifically, the GeoDetector model was used to detect spatial heterogeneity by measuring the explanatory power of different independent variables (*X*) on the dependent variable (*Y*). The calculation formula is expressed as follows:


$$q=1-\frac{\sum_{h=1}^{L}N_{h}\sigma_{h}^{2}}{N\sigma^{2}}.$$ (10)

Interaction detection is used to assess how the explanatory power of the dependent variable (*Y*) changes when two or more independent variables (*X*) interact (Table 1). This approach reveals whether multiple factors enhance, weaken, or independently contribute to the settlement pattern. The evaluation procedure involves overlaying two factors to generate a new q-value, which reflects their combined explanatory power. This value is then compared with the q-values derived from single-factor detection to determine the nature of the interaction. Table 1 presents the detailed evaluation results.

**Table 1:** Types of Interaction Detection and Validation Criteria.

Interaction type	Classification criteria
Nonlinear weakening	$q(X_{1}\cap X_{2})<\min(q(X_{1}),q(X_{2}))$
Single-factor nonlinear weakening	$\min(q(X_{1}),q(X_{2}))<q(X_{1}\cap X_{2})<\max(q(X_{1}),q(X_{2}))$
Two-factor enhancement	$q(X_{1}\cap X_{2})>\max(q(X_{1}),q(X_{2}))$
Independent	$q(X_{1}\cap X_{2})=q(X_{1})+q(X_{2})$
Nonlinear enhancement	$q(X_{1}\cap X_{2})>q(X_{1})+q(X_{2})$

In this study, raster data for each influencing factor were reclassified into five categories using the reclassification tool, with values assigned from 1 to 5. Settlement density was used as the dependent variable. These datasets were then compiled and input into the GeoDetector model to perform interaction detection and obtain the results.

## 3. Results

### 3.1. Spatiotemporal evolution analysis of settlements

#### 3.1.1. Spatial layout characteristics of settlements.

First, the ANN Index was employed to analyze the spatial patterns of settlements in Xilingol League across different periods (Table 2). The results show that settlement distributions consistently exhibited clustering throughout all periods. The nearest neighbor ratio increased from 0.27 to 0.62, while the *Z*-score rose from −14.03 to −31.04, indicating that the clustering intensity of settlements has continuously strengthened over time. The results were statistically significant at the 99% confidence level.

**Table 2:** Average Nearest Neighbor Index and Imbalance Index.

Time	Nearest proximity ratio	*Z*-score	*S* value
1915	0.27	−14.03	0.48
1933	0.32	−26.48	0.57
1949	0.37	−33.52	0.58
1958	0.38	−36.89	0.59
1970	0.59	−32.53	0.49
1980	0.62	−31.04	0.42

The Imbalance Index further revealed fluctuations in the spatial equilibrium of settlements. In 1958, settlement distribution was most concentrated (0.59), whereas by 1980, it reached its highest level of balance (0.42). This suggests that the spatial configuration of settlements in Xilingol League followed a wave-like trajectory of “balance—clustering—balance.”

**Fig 3:**
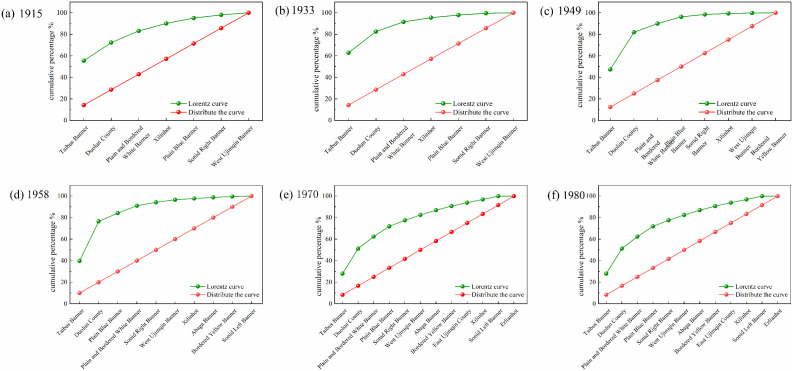
Lorenz curves of settlements for each period.

As illustrated in Fig 3, the Lorenz curve prior to 1958 deviated substantially from the line of perfect balance, reflecting the uneven distribution of settlements, which were primarily concentrated in the southern regions of Taibus Banner and Duolun County. After 1958, the number of settlements in other pastoral banners increased steadily, narrowing the gap between these areas and the agro-pastoral transitional zones. Consequently, the spatial distribution of settlements across the league became progressively more balanced.

#### 3.1.2. Evolutionary characteristics of settlement numbers.

**Fig 4:**
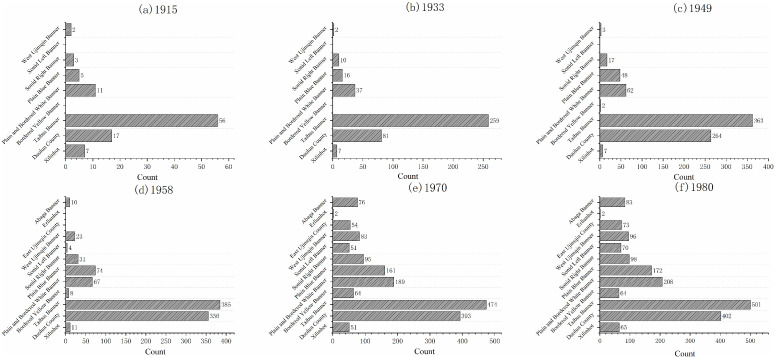
Settlement quantity for each period.

In the agro-pastoral transitional zone, Taibus Banner consistently recorded the highest number of settlements throughout the study period, reaching 501 settlements by 1980 (Fig 4). Among the pastoral banners, the Plain and Bordered White Banner had the largest number of settlements, totaling 208 in 1980. Notably, this banner borders Taibus Banner, suggesting that its settlement growth was strongly influenced by the radiation effect of settlement expansion from Taibus Banner, which further facilitated the spread of settlements into the pastoral areas.

**Fig 5:**
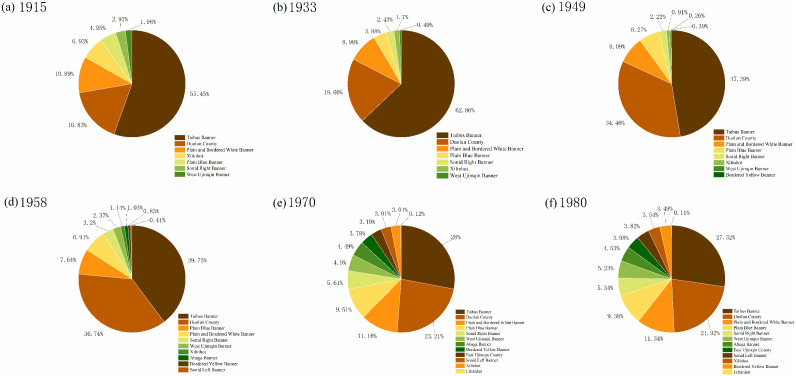
Proportion of settlement quantity for each period.

In terms of the proportion of settlements in agro-pastoral transitional zones (Fig 5), Taibus Banner and Duolun County together accounted for 72.28% of the league’s settlements in 1915, a share that further increased to 82.52% by 1933. After 1949, however, this proportion began to decline steadily, dropping from 81.85% to 76.47% in 1958, 51.21% in 1970, and 49.24% in 1980. This trend indicates that, following 1949, settlement growth in pastoral areas gradually accelerated, with the most significant proportional increase occurring between 1958 and 1970. During this period, the distribution of settlements across different banners became progressively more balanced, reflecting a positive trajectory toward equilibrium between pastoral and agro-pastoral transitional areas after 1958.

#### 3.1.3. Temporal evolution characteristics of settlement layout.

**Fig 6:**
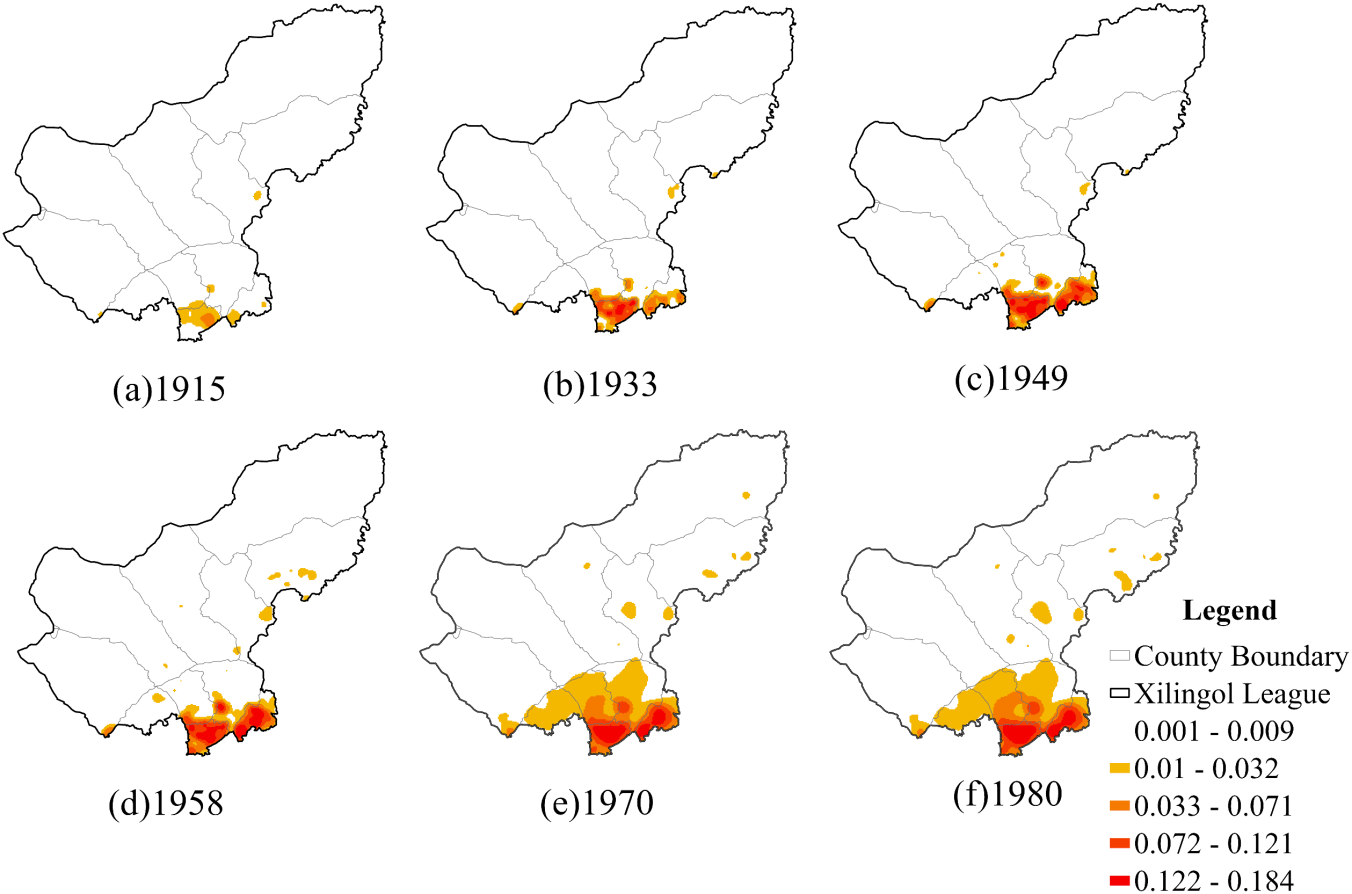
Kernel density distribution of settlements for each period. The map is produced based on the standard map from the Department of Natural Resources of Inner Mongolia (No. MS(2023)038), with no modification to the base map.

Using ArcGIS Pro, vector settlement points of Xilingol League during 1915–1980 were extracted. Based on major historical milestones, the settlement evolution process was divided into six historical periods, and the kernel density distribution for each period was generated (Fig 6). In 1915, settlement clustering first appeared in Taibus Banner, with relatively low intensity (below 0.032), concentrated mainly within its boundaries. By 1933, clustering intensity in Taibus Banner had increased markedly (above 0.072), and the clustering expanded eastward into the southern part of Duolun County. In 1949, both Taibus Banner and Duolun County exhibited high-density settlement clusters, while smaller, point-like high-intensity clusters also emerged in the southwest of Plain Blue Banner and Plain and Bordered White Banner. By 1958, settlement intensity in the four southern banners of Xilingol League further increased, with the clustering range largely consistent with that of 1949. Meanwhile, scattered low-intensity clusters appeared in the northern pastoral banners, indicating that settlement clustering was beginning to spread from the southern agro-pastoral transitional counties into the northern pastoral regions. In 1970, the clustering range expanded significantly northward, with noticeable clustering across Bordered Yellow Banner, Plain and Bordered White Banner, and Plain Blue Banner, though intensity remained relatively low. Scattered clusters in the northern pastoral areas also continued to expand. By 1980, the clustering scale had increased slightly compared to previous periods, with settlement expansion into pastoral areas becoming more evident. Overall, the kernel density analysis reveals a spatial pattern of strong, high-intensity clustering in the southern agro-pastoral transitional banners, alongside weaker and more scattered clustering in the northern and northeastern pastoral banners.

**Fig 7:**
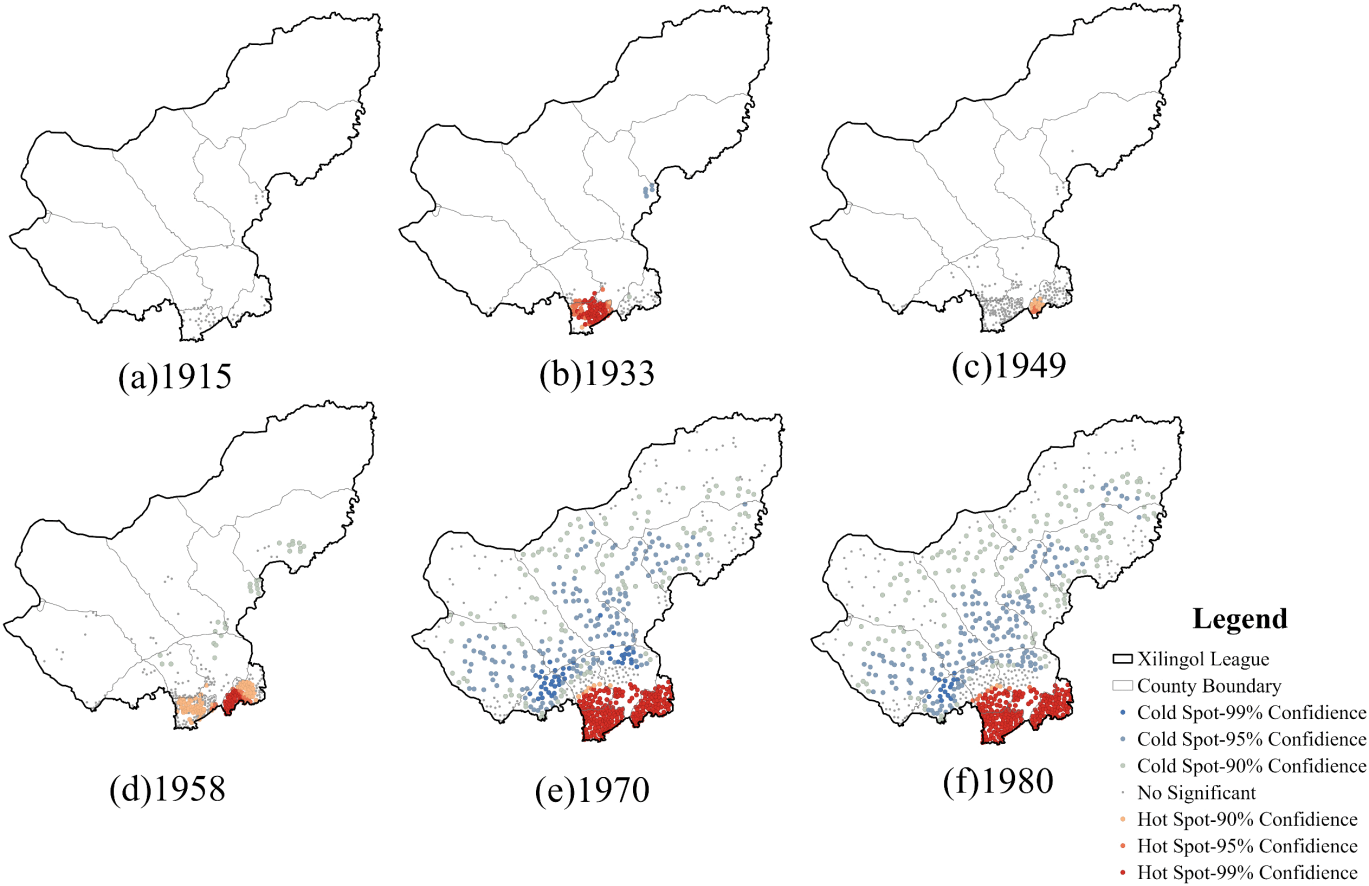
Cold and hotspot cluster distribution of settlements for each period. The map is produced based on the standard map from the Department of Natural Resources of Inner Mongolia (No. MS(2023)038), with no modification to the base map.

Analysis of the cold and hot spot clustering map (Fig 7) indicates that settlement hot spots in each period coincided with areas of high kernel density. The hot spots were concentrated in Taibus Banner and Duolun County, forming a distinct “south hot, north cold” pattern, with 42°N serving as the dividing line. In 1915, settlement development in Xilingol League was still limited, and no hot spot areas were observed. By 1933, a large hot spot area had emerged in Taibus Banner, and by 1958, this clustering further expanded into Duolun County. During the subsequent two periods, hot spot areas consistently remained within Taibus Banner and Duolun County, while the northern pastoral banners were characterized by cold spots or non-significant areas. Overall, after 1958, settlements began to emerge in the pastoral regions; However, due to distinct production modes and lifestyle patterns, no significant hot spot clusters were formed. In contrast, Taibus Banner and Duolun County, with their agro-pastoral mixed economies and relatively dense populations, consistently functioned as the settlement hot spot areas.

#### 3.1.4. The process of settlement mean center migration.

**Fig 8:**
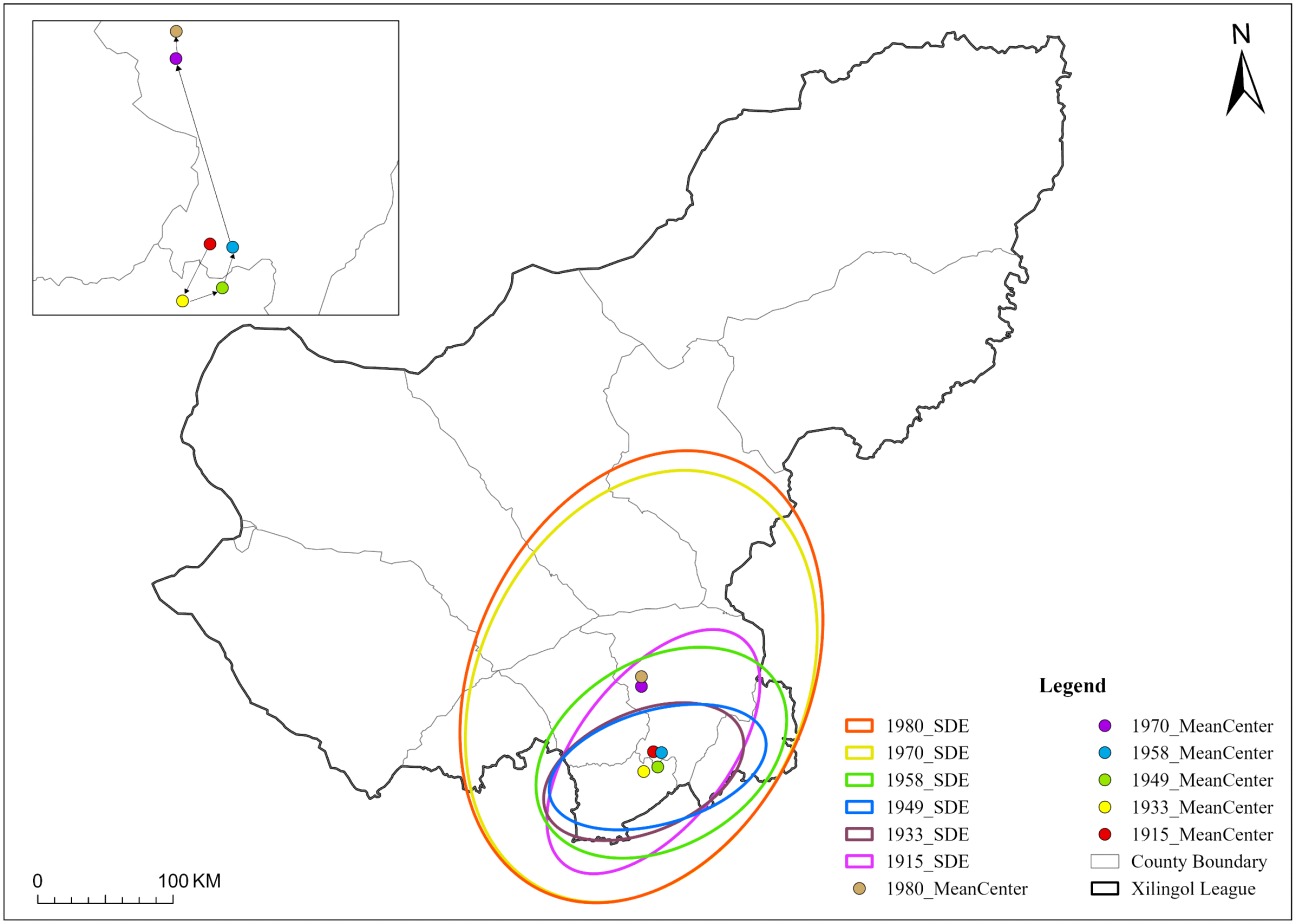
Standard deviation ellipse layout and movement of the mean center. The map is produced based on the standard map from the Department of Natural Resources of Inner Mongolia (No. MS(2023)039), with no modification to the base map.

Using the mean center and standard deviation ellipse tools to examine the directional dynamics of settlement expansion (Fig 8), the results reveal a “hook-shaped” trajectory of the settlement center, shifting from south to north. Before 1958, the settlement center fluctuated within a relatively stable range near the boundary between Taibus Banner and Plain Blue Banner. After 1970, however, it gradually migrated northwestward into Plain Blue Banner, indicating that prior to 1958 settlement movements were stable and short-range, whereas post-1958 expansion reflected a northward shift toward the pastoral banners, following an alternating process of incremental growth and spatial balancing. The standard deviation ellipse further illustrates this transformation (Table 3). Its area expanded markedly from 18,469.32 km^2^ to 68,094.89 km^2^, reflecting the significant enlargement of settlement scale. Spatial orientation also changed: the ellipse gradually rotated from an east–west to a northeast–southwest direction, while the tilt angle experienced a “horizontal-to-vertical” shift. These directional changes suggest that the development of settlements evolved from lateral expansion in the agricultural–pastoral mixed areas to longitudinal expansion into the pastoral zones, underscoring the progressive spread of settlement agglomeration toward the north.

**Table 3:** Standard deviation ellipse characteristic parameters.

Time	Area/Km^2^	Center coordinates	Rotation/°
1915	18469.32	$42^{\circ }10^{\prime }$ 10 N, $115^{\circ }37^{\prime }$ E	38.17
1933	10857.32	$42^{\circ }02^{\prime }$ 25 N, $115^{\circ }31^{\prime }$ 15 E	66.98
1949	10897.69	$42^{\circ }03^{\prime }$ 56 N, $115^{\circ }38^{\prime }$ 50 E	73.99
1958	21563.43	$42^{\circ }09^{\prime }$ 34 N, $115^{\circ }41^{\prime }$ 13 E	58.69
1970	63171.57	$42^{\circ }36^{\prime }$ 25 N, $115^{\circ }32^{\prime }$ 25 E	24.92
1980	68094.89	$42^{\circ }40^{\prime }$ 13 N, $115^{\circ }32^{\prime }$ 45 E	24.92

### 3.2. Factors influencing settlement distribution

#### 3.2.1. Single factor detection.

The formation of settlement spatial evolution is influenced by a wide range of factors. In this study, both natural and socio-economic dimensions are considered, with 14 representative indicators selected, including annual average temperature, annual precipitation, elevation, GDP, grassland area, cultivated land area, and newly added construction land (Table 4). Using the GeoDetector model, the spatial distribution of settlements in Xilingol League was analyzed to quantify the explanatory power of each indicator on settlement patterns. The q-statistic values derived from the analysis were ranked in descending order to indicate their relative influence on settlement layout: (*X*13) Agricultural acreage (0.6266)  >  (*X*11) Number of livestock (0.6215)  >  (*X*8) Population (0.4441) > (*X*6) Altitude elevation (0.3665) > (*X*12) Grassland area (0.3652) > (*X*4) Annual mean temperature (0.3511) > (*X*5) Average annual rainfall (0.1366) > (*X*14) New building area (0.1071) > (*X*10)/(*X*8) Total annual income of residents/Population (0.1014) > (*X*9) Agricultural and industrial output (0.0601) > (*X*3) Slope (0.015) > (*X*1) River distance (0.0109) > (*X*2) Aspect of slope (0.0023).

**Table 4:** The factors influencing the spatial distribution of settlements in Xilingol League.

Detection Factor		Serial Number	*q* statistic	*p* value
Natural environment	River distance/km	(*X*1)	0.0109	0.0056
	Aspect of slope/°	(*X*2)	0.0023	0.5392
	Slope	(*X*3)	0.0150	0.000
	Annual mean temperature/℃	(*X*4)	0.3511	0.000
	Average annual rainfall/mm	(*X*5)	0.1366	0.000
	Altitude elevation/m	(*X*6)	0.3665	0.000
Social economy	GDP/yuan	(*X*7)	0.1014	0.000
	Population/ten thousand	(*X*8)	0.4441	0.000
	Agricultural and industrial output value / ten thousand	(*X*9)	0.0601	0.000
	Total annual income of residents /yuan	(*X*10)	0.1014	0.000
	Number of livestock / ten thousand	(*X*11)	0.6215	0.000
	Grassland area/667 hectares	(*X*12)	0.3652	0.000
	Agricultural acreage/667 hectares	(*X*13)	0.6266	0.000
	New building area/m2	(*X*14)	0.1071	0.000

Based on the above results, the q-statistic values for socio-economic factors are consistently higher than those for natural environmental factors, indicating that the settlement layout in Xilingol League is more strongly shaped by socio-economic conditions. Historically, Xilingol League functioned as a traditional agro-pastoral society in which livestock husbandry was dominant and agriculture served as a supplement. However, the relatively high *q* values for socio-economic indicators—such as GDP, cultivated land area, and livestock numbers—demonstrate that these variables exert a stronger explanatory power on settlement evolution than natural environmental constraints. This finding reflects the steady progress of industrialization and modernization in the region, which has fostered a more diversified industrial structure. The advancement of industrial development has therefore played a decisive role in restructuring and guiding the spatial pattern of settlements in Xilingol League.

#### 3.2.2. Multi-factor detection.

**Fig 9:**
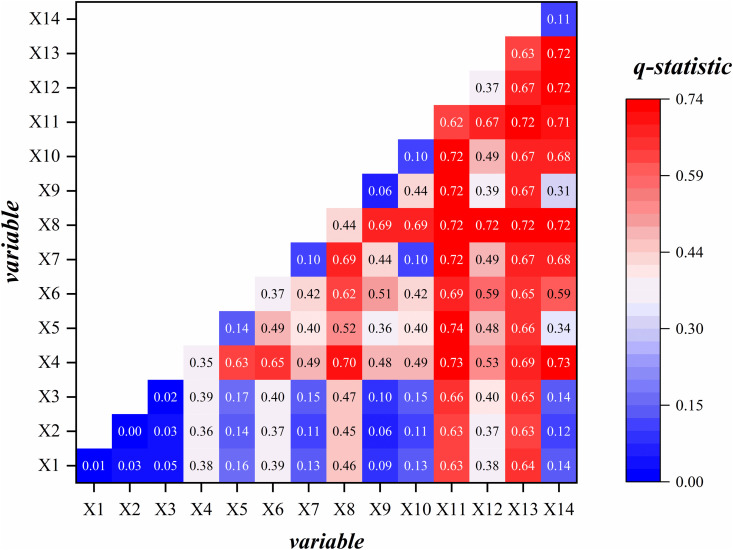
GeoDetector interaction factor results.

Another unique advantage of the Geodetector method is its ability to detect the interaction effects of two factors on the dependent variable. By incorporating the product term of two factors into the regression model, it becomes possible to test statistical significance and uncover the underlying mechanisms influencing settlement distribution in Xilingol League. This approach allows for a more comprehensive assessment of the relative importance of different indicators in the settlement distribution process, with the results visualized in the form of a heatmap (Fig 9). The interaction effects can be classified into two categories: bivariate enhancement and nonlinear enhancement. Compared with the explanatory power of individual factors, the combined influence of interacting variables markedly increases the spatial heterogeneity of the dependent variable. Results show that the interaction between the number of livestock and annual precipitation is the strongest (*q* = 0.74). This is followed by the interactions between the number of livestock and annual temperature, and between newly added construction area and annual temperature, both reaching 0.73. Moreover, factors such as livestock numbers, cultivated land area, population, and newly added construction area all exhibit strong interactive effects with other variables.

These findings highlight the deep-rooted environmental imprints of Xilingol League’s pastoral and agro-pastoral transitional zones. Population change, livestock numbers, and cultivated land area emerged as the dominant factors influencing settlement evolution during the study period. Their dynamics reflect three key trends: increasing population agglomeration, the expansion of agricultural cultivation, and the sedentarization of pastoral populations—together indicating that the traditional pastoral areas of Xilingol League are gradually transforming into modern agro-pastoral transitional zones.

## 4. Discussion

This study selects 1,834 village settlement points in Xilingol League between 1915 and 1980 to examine the process of settlement evolution and its driving factors. The results reveal that settlements in Xilingol League consistently exhibited a clustered pattern during the study period. However, the spatial layout of settlements underwent a transformation characterized by a wave-like process of “balance—clustering—balance.” Since 1915, settlements began to cluster in the southern part of the league, particularly in Taibus Banner and Duolun County, areas with relatively high elevation, favorable thermal conditions, higher precipitation, and denser river networks. From there, the clustering expanded northward in a fan-shaped pattern, though with gradually decreasing intensity. Regarding the driving forces behind settlement distribution, cultivated land area was identified as the single factor with the strongest explanatory power, while the interaction between livestock numbers and mean annual precipitation demonstrated the greatest combined influence. These findings are consistent with the conclusions of other scholars [45,52,53].

Xilingol League is located in an arid and semi-arid zone. In ancient times, nomadic peoples lacked the intrinsic motivation to transition toward an agrarian civilization. As a result, they were compelled to reduce the scale of farming, abandon sedentary pastoralism, and adopt a nomadic lifestyle [29], a mode of production that persisted throughout the feudal era. It was not until the early 20th century that substantial changes began to emerge. Facing massive indemnities and economic debts, the Qing government initiated the policy of “immigration and frontier cultivation” in 1902 to expand fiscal revenue. In 1915, the Republican government officially promulgated a decree on “Mongolian land reclamation.” Encouraged by these policies, large numbers of bankrupt farmers and merchants from Shanxi and Hebei migrated to the region, leasing or purchasing land and settling down. Consequently, Taibus Banner, Plain and Bordered White Banner, and Duolun County—areas geographically closer to Hebei—experienced significant population agglomeration. These three areas lie above 1,100 meters in elevation and are traversed by several perennial rivers, making them relatively favorable for early settlement. After 1933, with the establishment of the puppet Mongolian government, the “Livestock Production Expansion Plan” was introduced to protect grasslands. Clear boundaries were drawn between farmland in the counties and pastures in the banners, prohibiting arbitrary reclamation of grasslands and restricting the eastward expansion of agricultural populations. This also explains why, as shown in Fig 6, the mean settlement center shifted southwest during this period. The southern banners and counties thus began to serve as population agglomeration areas for agricultural migrants, marking the early urbanization of the agro-pastoral transitional zone. However, as human–land relations became increasingly strained, this also became the starting point for the expansion of settlement patterns. Land policies, a continental climate, and limited precipitation emerged as the key factors constraining the scale of settlements in pastoral areas before the founding of the People’s Republic of China [54].

Around 1949, in response to the needs of democratic reform and socialist transformation, the Inner Mongolia government implemented land reform in agricultural zones and democratic reform in semi-agricultural and pastoral as well as pastoral areas. Large estates and ranches were redistributed, stimulating the production enthusiasm of impoverished populations. From 1952 onward, mutual aid groups in pastoral areas and agricultural cooperatives proliferated. Pastures were gradually transformed into concentrated agricultural land, and settlements began expanding into surrounding banners [55,56]. After 1958, the People’s Commune Movement and the Great Leap Forward triggered extensive reclamation of grasslands. Communities sought fertile pastures with reliable water resources for farming, established large-scale state farms, and engaged in widespread land cultivation. The commune system concentrated both population and production resources, becoming a crucial driver for the emergence of settlements in pastoral areas and marking the starting point of sedentarization.

Between 1970 and 1980, the turbulence of the Cultural Revolution and the “sent-down youth” movement brought large numbers of urban youths into pastoral regions. During this period, slogans such as “agriculture must advance, animal husbandry must yield” were translated into concrete policies, while rural areas saw an influx of young labor. This led to another wave of grassland reclamation, further confirming the northward shift of the mean settlement center and kernel density intensity as shown in Fig 6. Institutional changes, population clustering in pastoral areas, and the expansion of cultivated land thus became the main factors influencing settlement transformation between 1949 and 1980.

The agro-pastoral ecotone serves as a buffer zone between farming and herding systems. Traditional definitions of the agro-pastoral ecotone often lack quantitative precision, making it difficult to fully capture its spatial distribution characteristics [57]. In this study, it was found that the 42°N latitude line not only delineates the cold and hot spot areas of settlements in Xilingol League but also coincides with the 400 mm precipitation line and the boundary between monsoon and non-monsoon zones. In recent decades, northern China’s agro-pastoral ecotone has experienced rising temperatures, with land-use boundaries shifting northwestward [58]. Although the southern part of Xilingol League lies at higher elevations compared with the pastoral zones, its thermal and precipitation conditions favor agricultural activities. This geographic line has thus become the boundary between agro-pastoral transitional areas and purely pastoral regions. Differences in production modes have also led to divergent settlement patterns: the agro-pastoral transitional zone experienced more rapid urbanization and population concentration, whereas the pastoral regions, though sparsely populated, began modern sedentarization largely in response to policy interventions.

The formation of settlements has been influenced by a combination of natural geography, cultural history, ecology, and other factors. Settlements and arable land constitute the core of the rural human–land system, underpinning agricultural, economic, and social structures [59]. During the study period, Xilingol League was still in the early stages of urbanization. Its settlement evolution process supports the findings of scholars that transportation conditions, economic development, residents’ income, and the emergence of new housing areas all influence land-use change and rural settlement patterns [60]. In particular, the changes in arable land and grassland areas exerted an amplifying effect on other factors [49]. Beyond these quantifiable factors, historical land and population policies directly shaped demographic changes and arable land expansion, serving as a crucial driver of settlement formation and pastoral urbanization in Xilingol League.

From the perspective of historical geography, this study applies GIS techniques to analyze the transformation of pastoral areas into agro-pastoral ecotones in Xilingol League before the reform era, providing methodological insights into the study of settlement evolution in northern China during periods without remote sensing imagery. Due to multiple adjustments of administrative divisions in Xilingol League, the recorded establishment times of some settlements remain somewhat ambiguous; However, this does not affect the overall trajectory of settlement evolution. Future research will further supplement the analysis with multi-language historical sources. In addition, the processes of urbanization and industrialization over the past decades have introduced certain ecological challenges. Rather than adopting a nation-centered critique of modernization, greater attention should be given to implementing effective ecological protection policies while also meeting the practical needs of regional modernization. How to avoid unsustainable modes of interaction between local agriculture and animal husbandry that may exacerbate ecological risks will be a key focus of subsequent research.

## 5. Conclusion

From 1915 to 1980, settlements in Xilingol League exhibited an overall clustered distribution, yet their spatial pattern remained uneven. The ANN value increased from 0.27 to 0.62. The settlement pattern experienced a fan-shaped expansion from the agro-pastoral banners in the south toward the pastoral banners in the north, with clustering intensity diminishing as the number of settlements increased, while in pastoral areas settlements tended to cluster in scattered forms around central towns. The distribution pattern was demarcated by the 42°N line, with a “hot south–cold north” spatial structure that simultaneously marked the boundary between the agro-pastoral ecotone and the pastoral zone, as well as the transition between urbanization and sedentarization. During the study period, socioeconomic factors were the primary drivers of settlement evolution. Expansion of arable land (0.6266), changes in livestock numbers (0.6215), and population growth (0.4441) collectively promoted the sedentarization process in Xilingol League. Moreover, variations in livestock numbers were significantly influenced by mean annual precipitation (0.74) and mean annual temperature (0.73). In essence, the transformation of settlement patterns reflected the northward shift of the agro-pastoral ecotone, representing the initial stage of sedentarization and modernization in the pastoral areas.

## Supporting information

S1 File Boundary of China. (SHX)

S2 File Boundary of Inner Mongolia. (SHX)

S3 File Boundary of xinligol. (SHX)

S1 Data Data. (XLSX)

S4 File Settlements of Xinligol. (ZIP)
