# Correction: Spatial and temporal pattern evolution and driving characteristics of rural settlements in Xilingol League from 1915 to 1980

**DOI:** 10.1371/journal.pone.0357393

**Published:** 2026-08-31

**Authors:** Sen Mu, Jianghong Zhen, Chun Xi

In the Funding statement, the grant numbers from the funder National Natural Science Foundation of China and Natural Science Foundation of Inner Mongolia Autonomous Region are listed incorrectly. The correct grant numbers are: 42361029 and 2025LHMS04008.
